# Distinct lipid membrane interaction and uptake of differentially charged nanoplastics in bacteria

**DOI:** 10.1186/s12951-022-01321-z

**Published:** 2022-04-15

**Authors:** Shang Dai, Rui Ye, Jianxiang Huang, Binqiang Wang, Zhenming Xie, Xinwen Ou, Ning Yu, Cheng Huang, Yuejin Hua, Ruhong Zhou, Bing Tian

**Affiliations:** 1 College of Life Sciences, Department of Physics, Institute of Quantitative Biology, Zhejiang University, Hangzhou, China; 2 Department of Chemistry, Columbia University, NY 10027 New York, USA

**Keywords:** Nanoplastics, Differentially charge, Lipid membrane, Uptake, Bacteria

## Abstract

**Background:**

Nanoplastics have been recently found widely distributed in our natural environment where ubiquitously bacteria are major participants in various material cycles. Understanding how nanoplastics interact with bacterial cell membrane is critical to grasp their uptake processes as well as to analyze their associated risks in ecosystems and human microflora. However, little is known about the detailed interaction of differentially charged nanoplastics with bacteria. The present work experimentally and theoretically demonstrated that nanoplastics enter into bacteria depending on the surface charges and cell envelope structural features, and proved the shielding role of membrane lipids against nanoplastics.

**Results:**

Positively charged polystyrene nanoplastics (PS-NH_2_, 80 nm) can efficiently translocate across cell membranes, while negatively charged PS (PS-COOH) and neutral PS show almost no or much less efficacy in translocation. Molecular dynamics simulations revealed that the PS-NH_2_ displayed more favourable electrostatic interactions with bacterial membranes and was subjected to internalisation through membrane penetration. The positively charged nanoplastics destroy cell envelope of Gram-positive *B. subtilis* by forming membrane pore, while enter into the Gram-negative *E. coli* with a relatively intact envelope. The accumulated positively charged nanoplastics conveyed more cell stress by inducing a higher level of reactive oxygen species (ROS). However, the subsequently released membrane lipid-coated nanoplastics were nearly nontoxic to cells, and like wise, stealthy bacteria wrapped up with artifical lipid layers became less sensitive to the positively charged nanoplastics, thereby illustrating that the membrane lipid can shield the strong interaction between the positively charged nanoplastics and cells.

**Conclusions:**

Our findings elucidated the molecular mechanism of nanoplastics’ interaction and accumulation within bacteria, and implied the shielding and internalization effect of membrane lipid on toxic nanoplastics could promote bacteria for potential plastic bioremediation.

**Graphical Abstract:**

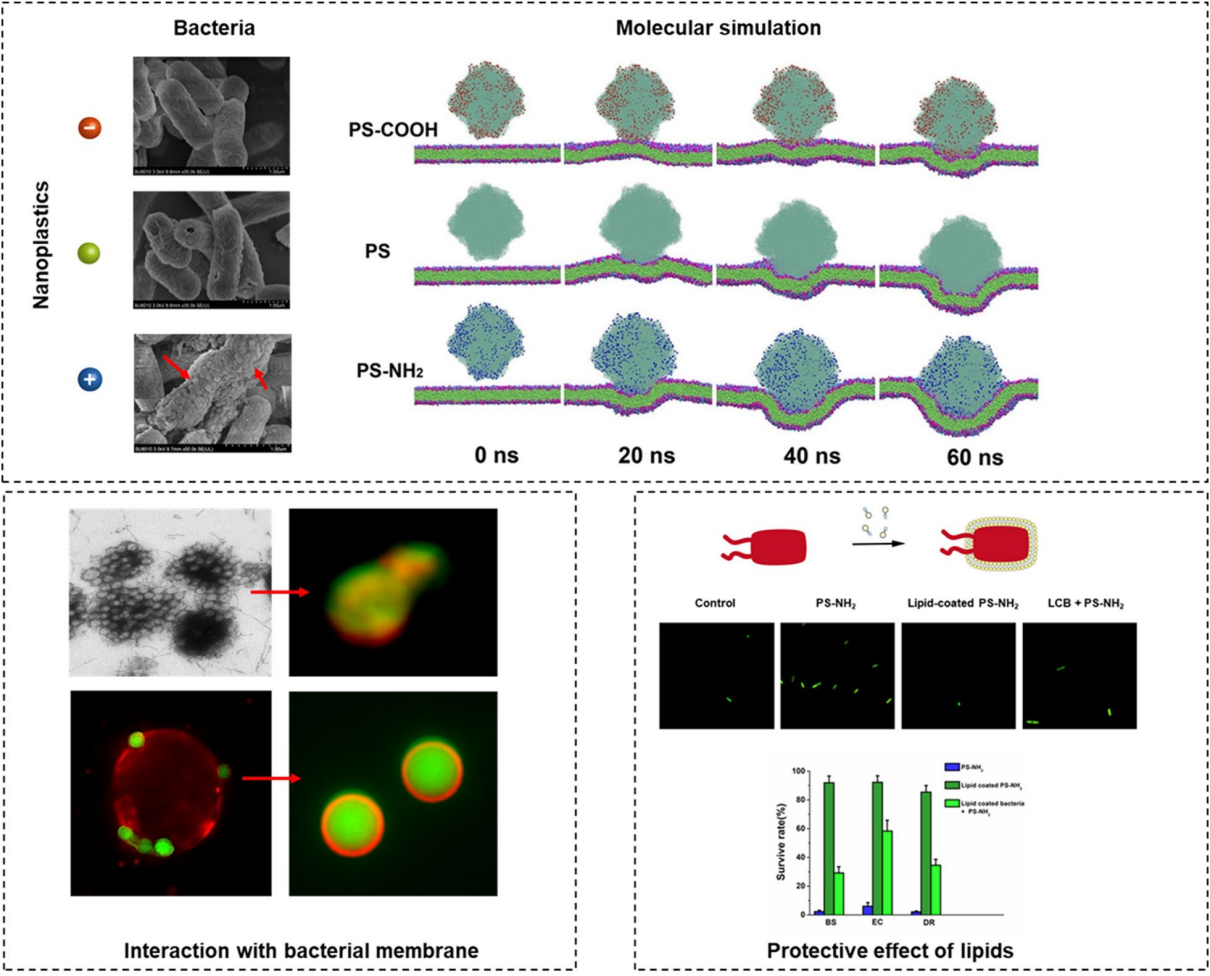

**Supplementary Information:**

The online version contains supplementary material available at 10.1186/s12951-022-01321-z.

## Background

The ever-increasing plastics are widely used across many fields that include manufacture, agriculture and everyday essentials, with the annual production of plastics exceeding 360 million tons in 2018 [1, 2]. Plastic wastes cause long-lasting pollution in oceans, lakes and soils of all continents [3–5]. Large plastic debris can be fragmented into microplastics (1 μm to 5 mm in size), submicroplastic (100 nm to 1 μm in size) and further decomposed into nanoplastics (1–100 nm in size) due to sunlight radiation and slow biodegradation [6–8]. Nanoplastics are distributed in the living environment [9–11]. Daily supplies, such as tea bags and 3D printers, are also sources of nanoplastics 12–14]. In the North Atlantic, microplastics are also digested into nanoplastics by marine organisms [15, 16]. The remarkable persistence and low biodegradable features of plastics, especially small-sized plastics, have recently raised pollution and health concerns globally [17]. Large-sized plastics could not pass through the physical barriers of intact cell envelope to enter cells due to size limitation. However, the nanoplastics can translocate into cells and accumulate in plant and animal tissues [18–21]. Polystyrene (PS) is a hazardous species of plastics and contains a high ratio of aromatic components; its fragments have adverse effects on living organisms [19]. PS particles are easy to be synthesized, labelled and characterized. [20]Plastic particles are possibly surface-charged by exposure to physical or chemical factors under various environmental conditions, such as light irradiation and oxidation [22, 23]. For instance, the microplastics acquire negative charges in pH 8.2 seawater due to the formation of electronegative ester carbonyl and ketone groups,[23] which can be further modified to form positively charged particles with the addition of amine groups. Charged PS nanoplastics accumulate and inhibit *Arabidopsis thaliana* growth and development [21]. In aquatic and terrestrial animals, PS nanoplastics can accumulate in organs and cells through the food chain, which cause oxidative stress, inflammation, immune dysfunction, neurotoxicity, neoplasia, changes in metabolism and energy homeostasis [24–27]. The principal route of nanoparticles’ entry into animal cell is via endocytosis [28]. For most gold nanostructures, the internalization mechanism in animal cell is considered to be receptor-mediated endocytosis [29]. For silica nanoparticles, its internalization was mediated by the clathrin-independent dynamin 2 [30] [31].Although recent studies have evaluated the distribution and accumulation of nanoplastics in plants and animals,[20, 21, 24, 25, 27, 31] little is known about the interactions of nanoplastics with bacteria, which are the basic living component of ecosystem at the lowest trophic level of the food chain.

Bacteria are ubiquitously distributed and they play an indispensable role in the ecosystem cycle. Bioremediation of plastic debris using bacteria is a promising technique [32–34]. Recently, a bacterium termed *Ideonella sakaiensis* was isolated; it is an efficient degrader to assimilate plastics (poly[ethylene terephthalate]) [34]. However, biodegradation is not yet a practical remediation nor a proven recycling strategy, partly because very few microbial species were found to be useful in plastic biodegradation despite many years’ exploration, and partly because there is a serious lack of understanding on the molecular mechanism of the interaction between nanoplastics and the bacteria. Hence, it is of great significance to understand the adsorption, transformation and potentially the degradation of nanoplastic substrates in bacteria. Moreover, recent research have showed that nanoplastics promote toxic microcystin release from *Microcystis aeruginosa*, which is the dominant species causing cyanobacterial blooms and posing a threat to both humans and the ecosystem [35]. Bacteria can take up metal and carbon nanomaterials,[36, 37] thereby indicating the possibility of uptake of nanoplastics by bacterial cells. Understanding the interactions of nanoplastics with bacteria is crucial for the risk assessment and ecosystem cycle of the nanomaterials. Unlike the plant and animal cells with few- layered envelopes, bacterial cells contain various multi-layered cell envelopes, which are classified into Gram-positive and Gram-negative bacteria based on Gram’s staining. The molecular mechanisms, however, are still largely unknown for the cellular internalisation process of differentially charged nanoplastics, particularly how exactly these nanoplastics translocate across bacterial cell membranes.

In this study, we aimed to determine how PS nanoplastics are translocated across the cell envelope of various bacteria, such as *E.coli* and *B. subtilis*, which represent the Gram-negative and Gram-positive bacteria, respectively, and *D. radiodurans* which has a thick multilayered cell envelope. To demonstrate the internalisation pathways of the nanoplastics into bacteria, we assessed the interaction process between PS nanoplastics with different surface charges and different bacteria using *in vitro* and *in vivo* experiments. Molecular details were further revealed using molecular dynamics simulation.

## Results and discussion

### PS nanoplastics with positive charges reduce bacterial viability

**Fig. 1:**
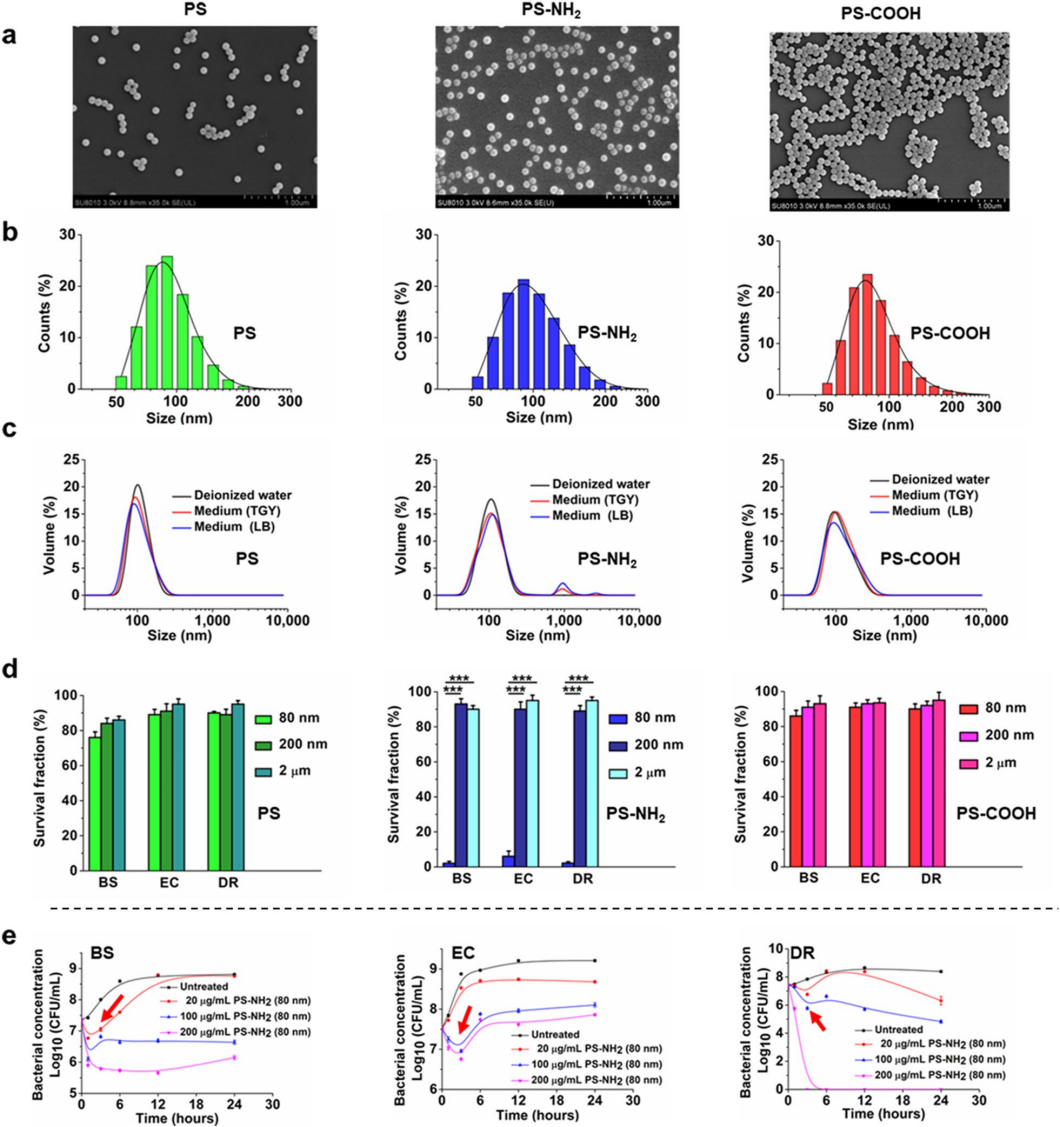
PS nanoplastics characterization and effects of the nanoplastics on the viability of bacteria. **a** SEM images of neutral PS, positively charged PS (PS-NH_2_), and negatively charged PS (PS-COOH) with the average size of 80 nm. Scale bars, 1 μm. **b** Size distribution of the PS, PS-NH_2,_ and PS-COOH as assessed using DLS in deionized water. **c** Stability of differentially charged PS nanoplastics in deionized water, TGY or LB medium. **d** Survive fractions of bacteria exposed to differently charged PS nanoplastics for 3 h compared with PS microplastics (200 nm or 2 μm in size). EC, *E*. *coli*; BS, *B*. *subtilis*; DR, *D. radiodurans*. *P* < 0.005; ***, significant difference (student’s *t*-test). **e** Growth curves of bacteria exposed to 80 nm PS-NH_2_ at different concentrations (20, 100, 200 µg/ml). Bacterial concentrations expressed as log_10_ (CFU/mL) of viable cells were measured at different growth times. Arrows indicate the drop in growth curves

PS particles with three types of surface charges (neutral, positive and negative charges) and three average sizes (~ 80 nm, ~ 200 nm and ~ 2 μm in diameter) were characterised and confirmed using scanning electron microscopy (SEM) and dynamic light scattering (DLS), respectively. The PS particles were well dispersed and stable in both deionised water and culture media (Fig. 1a–c and Additional file 1: Fig. S1). The zeta potentials of PS-COOH and PS-NH_2_ in deionised water (pH value of 7.0) were − 14.36 ± 0.55 and + 8.71 ± 1.08 mV, respectively. The PS particles with different sizes and charges were added to the culture of Gram-negative *E*. *coli*, Gram-positive *B*. *subtilis* and the extreme bacterium *D*. *radiodurans* and incubated for 3 h. The treatments of neutral and negatively charged PS nanoparticles (80 nm) as well as microparticles (200 nm or 2 μm) had no substantial influence on the surviving fractions of all these bacteria (Fig. 1d). However, the 80 nm positively charged PS (PS-NH_2_) caused a significant decrease in the surviving fractions of *B*. *subtilis* (97.7%), *E*. *coli* (94.0%) and *D*. *radiodurans* (97.9%) compared with the untreated control (*P* < 0.005), suggesting that the small-sized PS nanoparticles (~ 80 nm) with positive charges can remarkably reduce the viability of bacteria. By contrast, the 80 nm negatively charged PS (PS-COOH) and neutral PS did not inhibit bacterial growth even after long-term exposure (24 h) (Additional file 1: Fig. S2). A previous study showed that PS-NH_2_ could induce stronger cytotoxicity on mammalian cells than neutral and negatively charged PS nanoplastics [18]. In the plant *Arabidopsis thaliana*, positively charged PS nanoplastics inhibited plant growth more potently than the negatively charged ones [21]. Nevertheless, we discovered that the growth curve of bacteria exposed to 80 nm PS-NH_2_ displayed a transient drop at the beginning phase (0–6 h), after which growth gradually resumed (Fig. 1e). This finding indicated that stress response and recovery occurred in the bacterial population exposed to nanoplastics. To determine why bacterial growth can resume after PS-NH_2_ exposure, the nanoplastics within cell debris collected by centrifuge were added into the fresh culture of each bacterium, and cell growth was closely monitored (Additional file 1: Fig. S3). The recycled nanoplastics had a lower lethal effect on bacterial growth compared with primary PS-NH_2_, indicating that the impact of nanoplastics could be alleviated by components from sacrificial bacteria.

### Positively-charged PS nanoplastics penetrate the cell envelope and accumulate inside bacteria

**Fig. 2:**
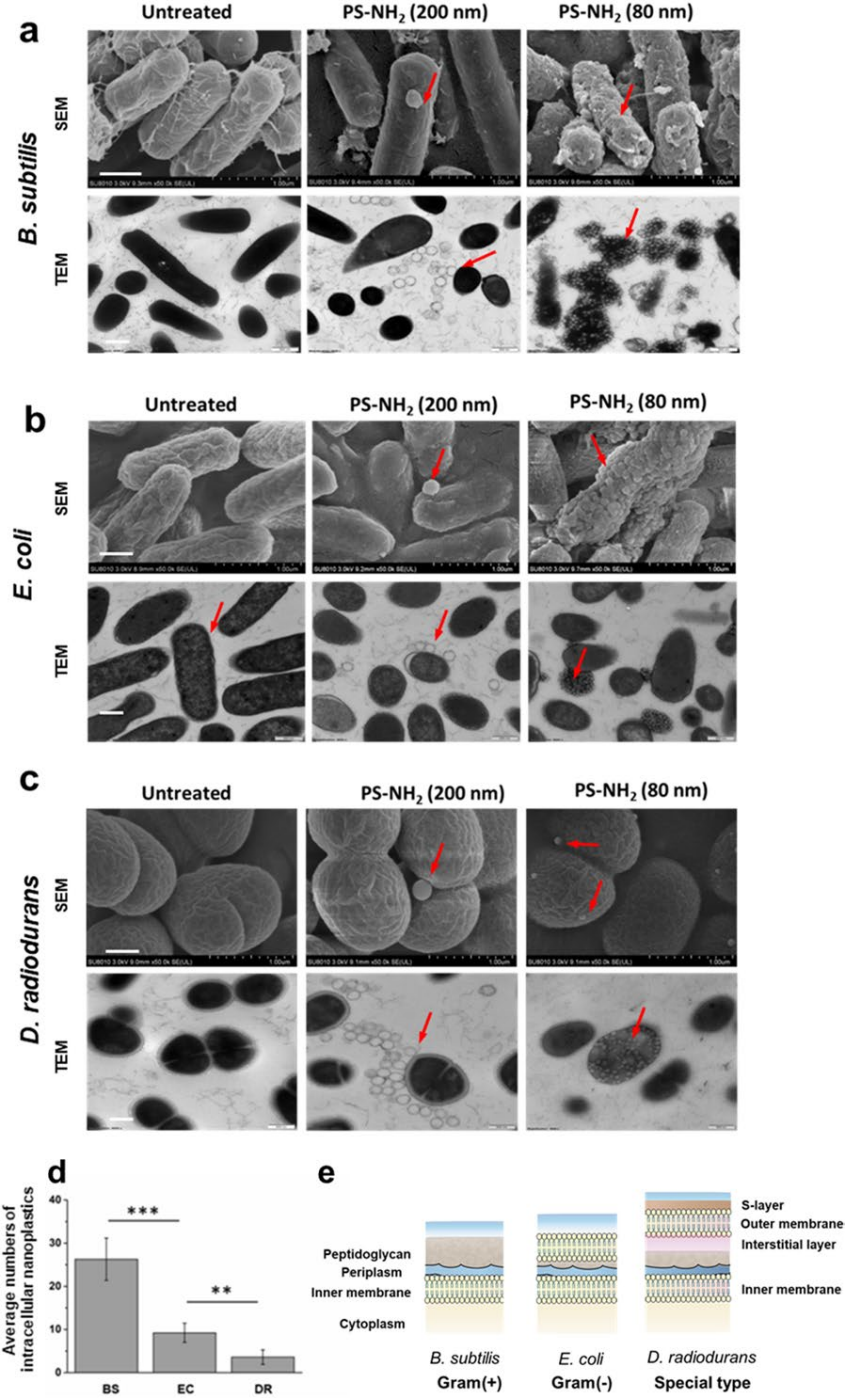
SEM and TEM images of the morphology of bacterial cells post-incubated with positively charged PS microplastics (200 nm) and nanoplastics (80 nm). *B*. *subtilis* (**a**), *E*. *coli* (**b**), and *D*. *radiodurans* (**c**) cells at OD_600_ of 1.0 were incubated with 100 µg/mL PS-NH_2_ (200 nm or 80 nm) for 3 h at 37 ^o^C or 30 ^o^C, respectively. Untreated (control), cells only treated with buffer solution. Red arrow, nanoparticles. Scale bar, 1 μm. **d** Relative level of intracellular nanoplastics per cell with intact morphology after incubated with 100 µg/mL PS-NH_2_ (80 nm) for 3 h, respectively. Average numbers of nanoplastics in the cell cross section from TEM images were used to represent the relative level of intracellular nanoplastics per cell. The error bars represent the standard deviations from five-independent statistics; **, *P* < 0.01; ***, *P* < 0.005, significant difference (student’s *t*-test). BS, *B*. *subtilis*; EC, *E*. *coli*; DR, *D. radiodurans*. **e** Schematic diagram of cell membrane structure of *B. subtilis*, *E. coli* and *D. radiodurans*

SEM images showed the 80 nm PS-NH_2_ embedded into the cell envelope of *E. coli* and *D. radiodurans*, whereas they punched holes (pores) on the envelope and then left pores on the cells of *B. subtilis* (Fig. 2a-c). Moreover, the results of fluorescent probe SYTOX uptake assay proved that the cell envelope of *B. subtilis* was damaged by the PS-NH_2_, leading to increased membrane permeability (Additional file 1: Fig. S4). The PS-NH_2_ nanoplastics accumulated tremendously and distributed widely across the cytoplasm of both *E. coli* and *D. radiodurans*, whereas they distributed mostly in the cellular periphery of *B. subtilis*, resulting in cell lysis with hollowed debris, as shown in TEM images (Fig. 2a-c and Additional file 1: Fig. S5). The strong action of PS-NH_2_ with pore formation on the outer envelope of Gram-positive *B. subtilis* is similar to that on animal macrophage cells, possibly because both cells have thin envelopes containing cytoplasm membrane [38]. Moreover, by counting the number of nanoplastics from cell cross section images of bacterial cells, we found that *B. subtilis*, which has a thinner envelope, accumulated more nanoplastics than *E. coli*. And *D. radiodurans* accumulated even fewer nanoplastics than *E. coli* (Fig. 2d). Compared with the Gram-positive bacteria, Gram-negative *E. coli* had an additional outer membrane containing lipopolysaccharide (Fig. 2e). The cell envelope of the extreme bacterium *D. radiodurans* consisted of at least five layers with a total thickness of 150 nm, namely, (i) cytoplasmic membrane, (ii) peptidoglycan-containing layer, (iii) interstitial layer, (iv) outer membrane and (v) surface layer containing hexagonally packed protein subunits [39]. The differential envelopes of bacteria might be responsible for the distinct translocation and distribution of the PS-NH_2_. The action of PS-NH_2_ on bacterial cells depends on the cell envelope structure of the bacteria.

Both the cationic and the anionic forms of many nanoparticles including PS nanoplastics, can be taken up by the animal cells [40–42]. However, no intracellular accumulation of negatively charged PS-COOH and very few neutral PS were observed to be embedded or inside the bacterial cells even after 12 h of exposure (Additional file 1: Fig. S6). Moreover, the large-sized PS-NH_2_ (200 nm) were found to be attached on the cell surface but not internalised inside the bacterial cell (Fig. 2). The translocation and interaction of PS nanoparticles of the same size depend on the surface charges of nanoparticles and cell envelope properties of the bacteria. Overall, our results demonstrated that positively charged nanoplastics with small sizes (< 80 nm) could efficiently translocate across cell envelopes and accumulate inside bacteria.

### Biophysical model of the PS nanoplastic–membrane interactions

**Fig. 3:**
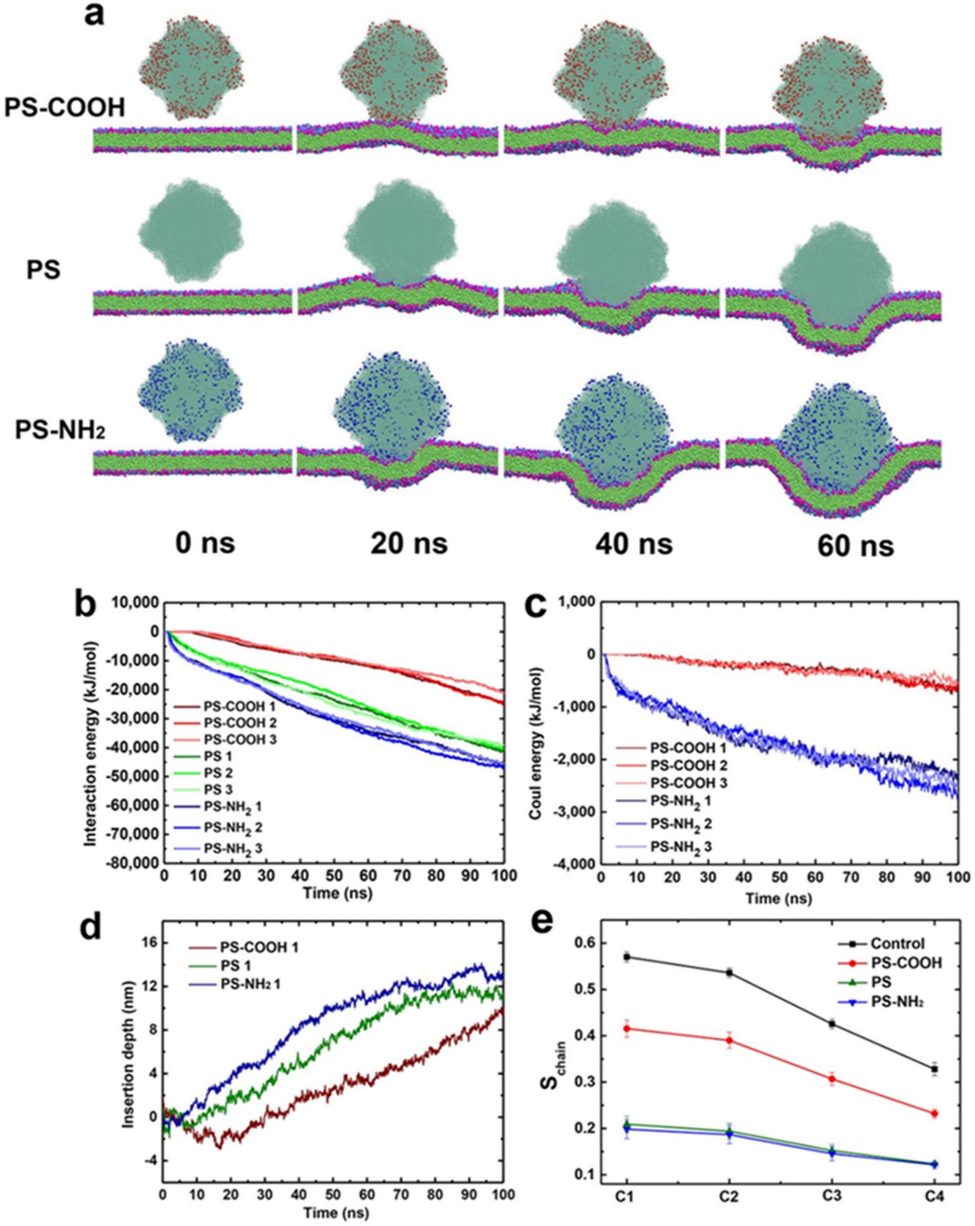
Molecular dynamics simulations of the interaction between differentially charged nanoplastics and the cytoplasm membrane. **a** Representative trajectories for the simulations of the binding of negatively-charged, neutral, and positively-charged nanoplastics (16 nm) with the mimic *E. coli* cytoplasm membrane **(**3:1 mol ratio of POPE:POPG**)**. POPE, 1-palmitoyl-2-oleoyl-sn-glycero-3-phosphoethanolamine; POPG, 1-palmitoyl-2-oleoyl -sn-glycero-3-phosphoryl glycerol. **b** Time evolution of the interaction energy (including vdW and Coul energies) between differentially charged nanoplastics and *E. coli* cytoplasm membrane. Three independent simulations were indicated with numbers. **c** Time evolution of Coul energies between differentially charged nanoplastics and the *E. coli* cytoplasm membrane. **d** Time evolution of insertion depth of differentially charged nanoplastics. **e** The lipid tail order in terms of chain order S_chain_

To better understand the mechanism underlying the translocation of nanoplastics into bacterial cells, we performed molecular dynamics simulations to study the detailed interactions of differentially charged PS nanoplastics with the cell membrane. The modelling details of the nanoplastics and membrane lipid bilayer were provided in the supporting information (Additional file 1: Figs. S7, S8 and S9). The membrane lipid bilayer was modelled with 3:1 mixed 1-palmitoyl-2-oleoyl-sn-glycero-3-phosphoethanolamine (POPE) – 1-palmitoyl-2-oleoyl-sn- glycero-3-phosphoryl glycerol (POPG) to resemble the cytoplasm membrane lipid composition of *E. coli*, in accordance with our previous studies [37]. The nanoplastics PS-COOH, PS-NH_2_ and neutral PS were initially placed 1.2 nm above the membranes, and three independent 100 ns simulations were performed for each type of nanoplastic. Figure 3a shows the snapshots taken from representative trajectories for the three types of nanoplastics at 0, 20, 40 and 60 ns, respectively. The penetration of the nanoplastics into the cytoplasm membrane was represented at two stages. At the first stage, the nanoplastics freely moved and then approached the membranes. More significant differences occurred at the second stage (t ≥ 20 ns). The insertion of the positively charged PS-NH_2_ was the most rapid among the PS nanoplastics, leading to membrane invagination (Fig. 3a, Additional file 1: Fig. S10), which coincided with the SEM images of cells exposed to PS-NH_2_ (Fig. 2). At t = 100 ns, the major portion of the PS-NH_2_ had entered the membrane. Eventually, the PS-NH_2_ nearly completely entered the cell membrane (see below). Moreover, the insertion depth of nanoplastics (defined in Additional file 1: Fig. S9a) increased in the order of PS-NH_2_ > PS > PS-COOH within the same simulation time, as shown in Fig. 3d and Additional file 1: Fig. S10.

Furthermore, we analysed the interaction energies of PS-COOH, PS and PS-NH_2_ with the cytoplasmic membrane (Fig. 3b). PS-NH_2_ exhibited the strongest interaction with the membrane, indicating that it may easily overcome the barrier from the interfacial water and the self-interaction among phospholipid molecules. High binding of positively charged particles with cytoplasm membrane can increase surface tension and deform the membrane [43]. Thus, PS-NH_2_ can translocate across the membrane more easily than PS-COOH and neutral PS. The entry of PS-COOH was difficult, as it had the weakest interacting energy with the membrane. Furthermore, we analysed the Coulombic interaction energies of PS-COOH and PS-NH_2_ (Fig. 3c) separately. The Coulombic interaction energy of positively charged PS-NH_2_ with the membrane was stronger than that of negatively charged PS-COOH, which is due to the negatively charged surface of the cell membrane. These results indicated the ready penetration of PS-NH_2_ was promoted by electrostatic attraction with the overall anionic phospholipid cell membrane. This is distinct from the almost no or much less permeation across lipid bilayer membranes by negatively charged or neutral nanoparticles [40, 41].

To further explore the effect of PS nanoplastics on the cytoplasm membrane, we calculated the lipid tail order in terms of the chain order parameter S_chain_ (as defined below). In the Martini model, the lipid tail of POPE and POPG was modelled by four hydrophobic beads,[44] namely the C1, C2, C3 and C4 beads (Additional file 1: Fig. S8a), and their tails were selected to calculate the order parameter at each bead site (chain order). The chain order parameter at each bead was then estimated by the function S_chain_=0.5<3cos^2^*θ*− 1> [45]. The *θ* is the angle between the bilayer normal and the orientation along the hydrocarbon chain, which is defined as the vector between the C_n_ and C_n+1_ (n=0, 1, 2, 3) beads (Additional file 1: Fig. S9b). The order parameter was averaged over all the membranes in all trajectory samples. The values of 1, −0.5 and 0 represent the perfect alignment, antialignment and random orientation,[46] respectively. As illustrated in Fig. 3e, all three types of nanoplastics reduced the lipid tail order as compared to the control group. The lipid tail order of membranes exposed to PS-NH_2_ and PS was much lower, implying that the membrane structure was considerably damaged, which was consistent with the respective cell morphology changes (Fig. 2 and Additional file 1: Fig. S5).

In addition, as the temperature rose, the translocation capacity of PS-NH_2_ into bacterial cells will enhance, which may be caused by the increase in the fluidity of the membrane. The simulation results were verified by our experiments, which showed that elevated temperatures increased the inhibition of PS-NH_2_ on bacteria (Additional file 1: Fig. S11). For all the above temperature simulations, the efficient translocation of PS-NH_2_ into bacterial cytoplasm membrane occurred via direct penetration promoted by electrostatic interaction with the anionic cell membrane, and it was distinct from the endocytic pathways adopted by animal kidney cells, which are facilitated by endocytic proteins, such as clathrin [47].

### Internalised PS-NH_2_ form lipid-coated nanoplastics

**Fig. 4:**
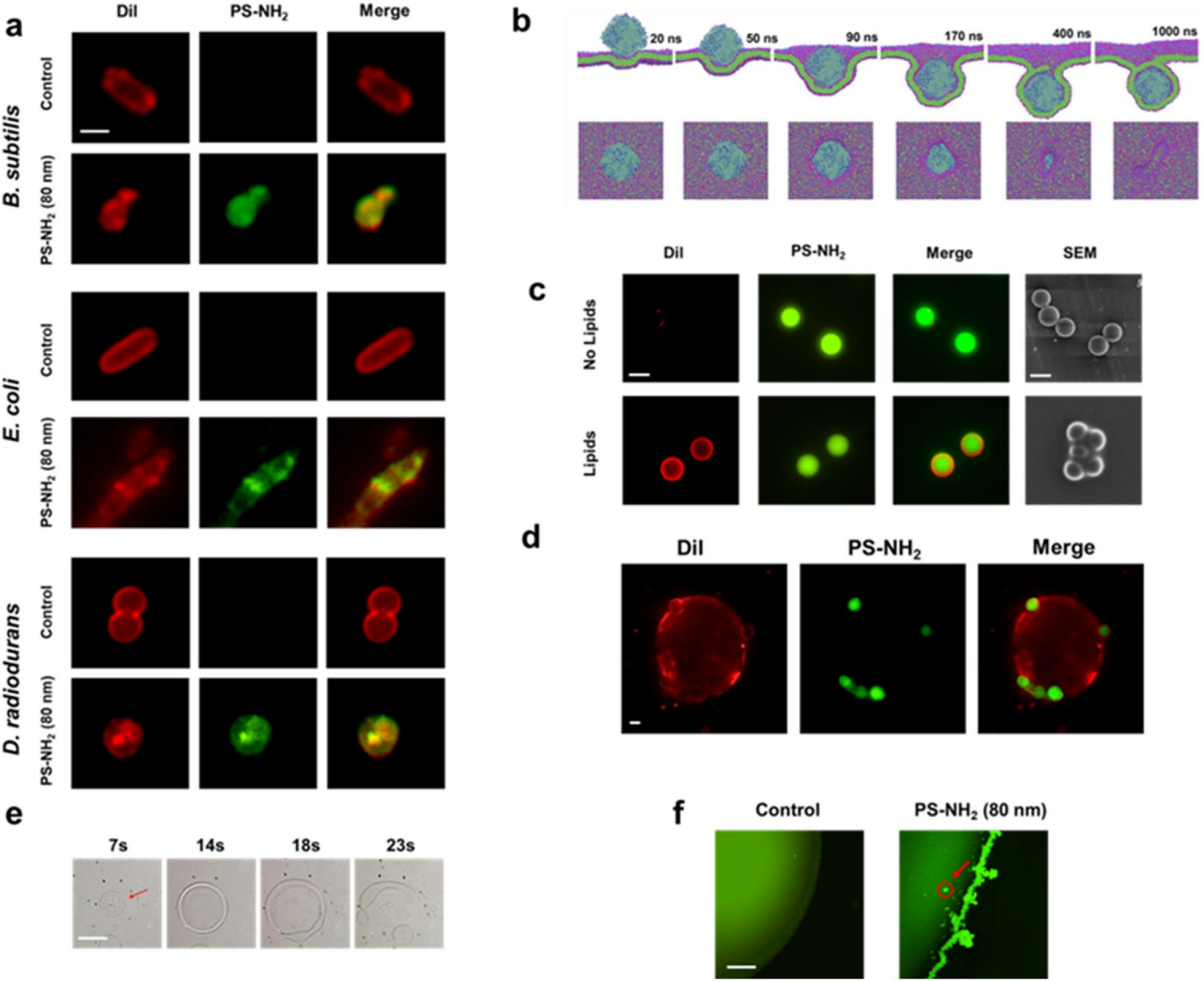
Internalized PS-NH_2_ formed lipids packing nanoplastics. **a** Confocal laser scanning microscopy on the localization and morphology of fluorescently labeled PS-NH_2_ (green) and membrane lipids (red) in bacteria following exposure to PS-NH_2_ (80 nm) for 3 h. Control, bacterial cells in the absence of PS-NH_2_. Merge, an overlap of the images of lipids stained by Dil and PS-NH_2_. Scale bars, 1 μm. **b** Molecular dynamics simulations of PS-NH_2_ (16 nm) uptake by the mimic membrane bilayers. Top panel, front view; Bottom panel, top view. **c** Confocal laser scanning microscopy of 2 μm PS-NH_2_ coated with lipids. The nanoparticles were incubated with or without a lipid mixture containing phospholipids in fatty acids. Dil dye was used for staining the phospholipid layer. SEM image was also recorded to show the different textures between the pure PS-NH_2_ and PS-NH_2_ coated with lipids. Scale bars, 2 μm. **d** Confocal laser scanning microscopy of the interaction and morphology of 2 μm PS-NH_2_ with a synthetic unilamellar liposome following incubation for 15 min. Scale bars, 2 μm. **e** Time course of morphology changes of the macro artificial lipid membrane by dripping 1 µL PS-NH_2_ (80 nm) from the top. The artificial lipid membrane (radius ≈ 0.5 cm) was constructed by dripping a drop of the mixture of phospholipids and fatty acids on the surface of deionized water in a plate (Additional file 1: Fig. S12). Scale bars, 5 mm. **f **Morphology changes of the artificial lipid membrane by dripping 1 µL PS-NH_2_ (80 nm) from the membrane boundary. Control, lipid membrane in the absence of PS-NH_2_. The red arrow indicates the internalized PS-NH_2_. Scale bars, 0.3 mm

We examined the location and morphology of PS-NH_2_ in bacterial cells and artificial lipid bilayer exposed to the fluorescently labelled nanoplastics through confocal laser scanning microscopy. Following exposure to PS-NH_2_ for 3 h, PS-NH_2_ (80 nm) labeled with green fluorescence was detected in the cytoplasm of each bacterium compared with the control (Fig. 4a). The PS-NH_2_ distorted the cell envelopes, and similar results were observed in TEM and SEM images (Fig. 2 and Additional file 1: Fig. S5). Moreover, co-location of the nanoplastics and lipid layer was detected as the merged images showed yellow fluorescence (Fig. 4a), suggesting that the PS-NH_2_ might be eventually coated with lipids obtained from cell membranes during its penetration across the cell envelope. Furthermore, the coarse-grained molecular simulations of the PS-NH_2_ uptake process by the membrane bilayers showed that the penetration of PS-NH_2_ led to gradual membrane invagination, the final internalisation of the lipid-coated nanoplastics around 1000 ns, and a finally closed transient pore (Fig. 4b, Additional files 2 and 3: Movies 1 and 2). To verify this nanoplastic-lipid-coating process more visually, a larger PS-NH_2_ (2 μm) labelled with green fluorescence was used to observe the interaction of the plastic particles with artificial lipid bilayer. The lipid bilayer was constructed by using phospholipids (3:1 of POPE:POPG) and fatty acids (4:1 of squalene:oleic acid). A clear lipid layer surrounding the nanoplastics was revealed by the strong red fluorescent ring-like entity, which was detected around the green PS-NH_2_ as compared to the bare PS-NH_2_ without lipid interaction (Fig. 4c). This finding demonstrated that the PS-NH_2_ can be easily coated with available lipids. Furthermore, the *in vitro* synthetic unilamellar liposome solution formatted by the ultrasonication of the phospholipids and fatty acids was incubated with the 2 μm PS-NH_2_ for 15 min and then examined using the fluorescence microscope. The PS-NH_2_ could approach and penetrate the lipid bilayer, thereby forming a lipid-coated PS-NH_2_ in the liposome (Fig. 4d). The higher the hydrophobicity of a nanoparticle, the greater the affinity of the particle to the bacterial membranes [48]. Previous studies also presented that Au nanoparticles modified with hydrophobic polyampholytes could be adsorbed onto DOPC liposomes [49]. Thus, the PS-NH_2_ was bound to the liposome via electrostatic and hydrophobic interactions, leading to the formation of lipid-coated PS-NH_2_.

To verify the surface tension that the nanoplastics impose on membranes, a macro artificial lipid membrane (radius ≈ 0.5 cm) was constructed by dripping a drop of the mixture of phospholipids and fatty acids on the surface of deionised water in a plate. 1 µL PS (80 nm) with different charges was dripped from the top or the side of the artificial lipid membrane, as shown in the diagram (Additional file 1: Fig. S12). The action of PS-NH_2_ induced surface tension on the membrane with increasing treatment time and led to complete membrane collapse at 23 s (Fig. 4e). However, the neutral PS and PS-COOH did not affect much to the membrane structure (Additional file 4: Movies 3). Similar morphological deformation was observed when the PS-NH_2_ was dripped from the side of the membrane. The PS-NH_2_ accumulated and definitely penetrated the lipid membrane (Fig. 4f). Positively charged PS could efficiently internalise into bacteria cells via a penetration and subsequent lipid coating.

### Lipid coating mediates the interaction between PS-NH_2_ and bacterial cells

**Fig. 5:**
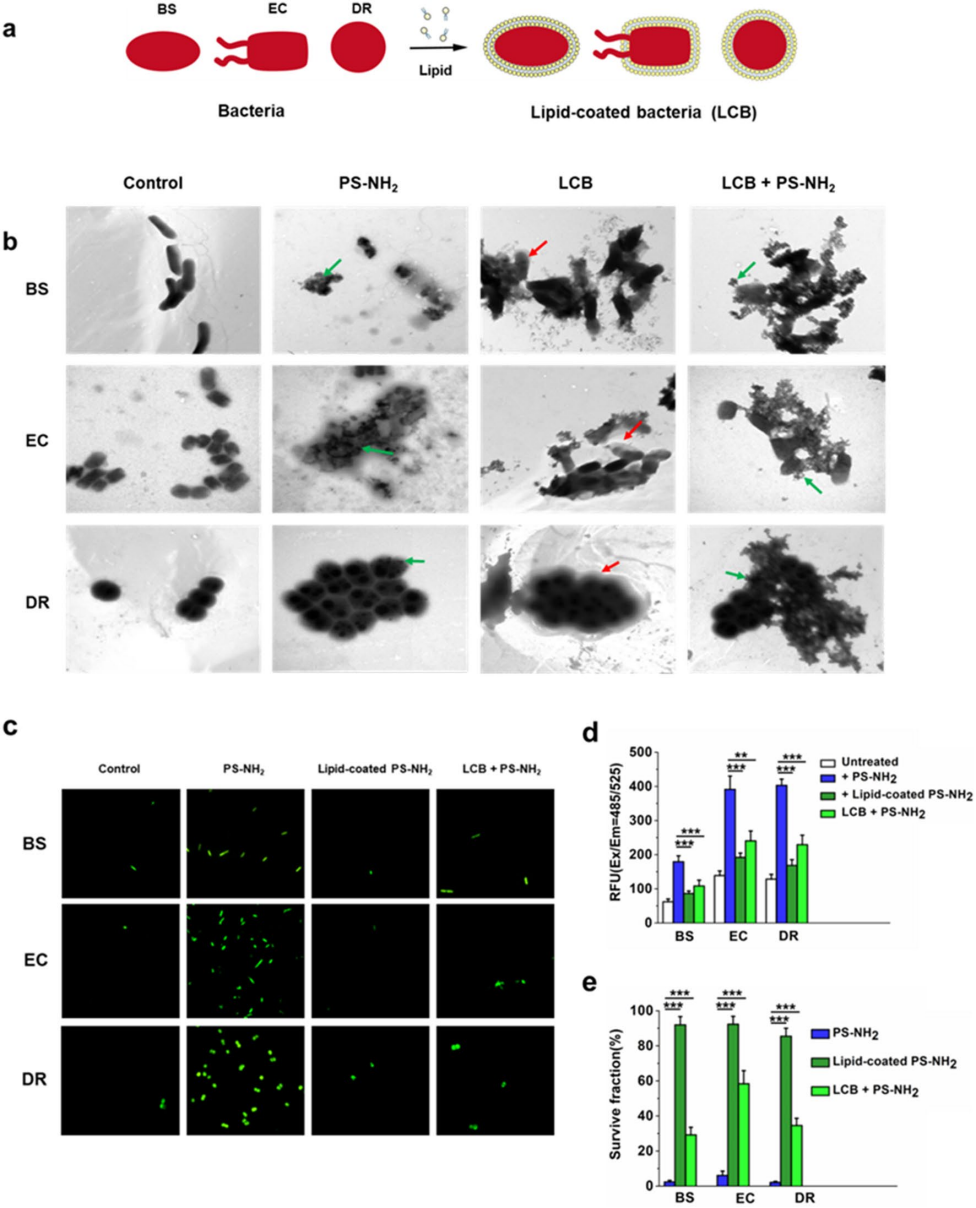
Impact of PS-NH_2_ on bacteria was alleviated by coating with a lipid layer. **a** Schematic diagram of the preparation of lipid-coated bacteria (LCB) by biointerfacial supramolecular self-assembly. The bacteria cells were wrapped up with lipid layers. **b** TEM images of the lipid-coated bacteria incubated with or without the PS-NH_2_ (100 µg/ml) for 3 h. TEM images of uncoated bacteria were used as the controls. Nanoplastics and membrane coat was indicated by the green arrow and red arrow, respectively. **c**, **d** Intracellular ROS level detected by green fluorescence signals in bacteria exposed to non-treatment (control), PS-NH_2_ (100 µg/ml), and PS-NH_2_ coated with lipids for 3 h, respectively. LCB + PS-NH_2_, LCB treated with PS-NH_2_ for 3 h. The nanoparticles and bacteria were incubated with or without a lipid mixture containing phospholipids and fatty acids. **e** Survive fractions of different bacteria exposed to PS-NH_2_ (100 µg/ml) or PS-NH_2_ coated with lipids, and survive fractions of respective LCB exposed to PS-NH_2_ for 3 h. The error bars represent the standard deviations from five-independent statistics; *, *P* < 0.05; **, *P* < 0.01; ***, *P* < 0.005, significant difference (student’s *t*-test). LCB + PS-NH_2_, lipid coated bacteria (LCB) treated with PS-NH_2_ for 3 h. EC, *E*. *coli*; BS, *B*. *subtilis*; DR, *D. radiodurans*

To verify the effect of the lipid coating on the strong interaction between PS-NH_2_ and bacterial membrane, the bacterial cells were “buffered” with an artificial lipid membrane by vortexing the cells with dioleoylphosphatydic acid (DOPA) and cholesterol in calcium phosphate buffer[50] (Fig. 5a). These lipid-coated bacteria (LCB) became more isolated from the nanoplastics than the control bacteria (Fig. 5b), confirming the shielding effect of lipid coating. The PS-NH_2_ (80 nm) exposure triggered the generation of intracellular ROS with decreased survival in the bacteria (Fig. 5c–e), thereby indicating a stress response that corresponded to the transient drop in growth at the early exposure phase (0–6 h) (Fig. 1e). However, little ROS signal was detected in both the bacteria exposed to the lipid-coated PS-NH_2_ and in the LCB exposed to the PS-NH_2_ (Fig. 5a and d), which resulted in the significantly higher survival of the respective bacteria compared with the bacteria exposed to the PS-NH_2_ (*P* < 0.05) (Fig. 5e). This result suggested that the detoxification of the PS-NH_2_ was achieved via the lipid coat formation, which prevented the interaction between nanoplastics and bacterial cells. This finding may explain the observed growth resumption in the later exposure phase (Fig. 1e). The accumulated positively charged nanoplastics in cells conveyed cell disruption. Subsequently released nanoplastics with lipid coating from the broken cells (sacrificial cells) are less damaging to other cells. Thus, lipid coating on nanoplastics might be a natural strategy of bacteria to protect themselves against the attack of primary nanoplastics.

## Conclusions and implications

Our results demonstrated that the uptake of PS nanoplastics by bacteria depend on the surface charge properties of nanoplastics and bacterial cell envelope structures. The positively charged PS-NH_2_ nanoplastics demonstrated a more efficient translocation via a membrane penetration and subsequently lipid-coating, which resulted in accumulation inside bacteria, whereas negatively charged PS-COOH and neutral PS displayed almost no or much less translocation. The interaction of PS-NH_2_ with membrane caused cell disruption and induced oxidative stress. The lower lethality of the lipid-coated nanoplastics might be a natural strategy of bacteria to shield the actions of positively charged PS nanoplastics. Our findings elucidated the interaction mechanism of nanoplastics with bacteria, thereby providing new insights to the physiologically relevant behaviour of nanoplastics with environmental bacteria and intestinal microflora. And our biophysical model of the nanoplastic–membrane interactions revealed a distinct translocation mode of charged nano-PS, which could also be a feasible method to analyze the interaction between cell membrane and other species of nanoplastics, such as polyvinyl chloride (PVC) and polyethylene glycol terephthalate (PET). Further studies on the translocation and uptake of other plastic polymers, such as polyethylene and polypropylene, are needed to better understand the impacts of various plastic types on bacterial cells. The actions of nanoplastics with other shapes and sizes that closely mimic the plastic population in the natural environment are also needed for further investigation. Shielding strong interactions by lipid-coating has health implications, and the uptake of nanoplastics can be controlled by regulating microenvironmental lipids. These findings also indicated potential strategies for efficient plastic bioremediation using engineered plastic-degrading bacteria through the improvement of the bioavailability of nanoplastics by either changing the surface charges or tunning the lipid-coating on nanoplastics, thereby maximising the use of bacteria to assimilate and recycle environmental nanoplastics.

## Materials and methods

### Nanoplastics and bacteria materials

Stock solutions of fluorescently labeled PS, PS-NH_2_ and PS-COOH particles were custom synthesised from Da’e Scientific Co., Ltd. (Tianjin, China). The fluorescent dye (NB or NBD-Cl) was chemically incorporated into the polymer network of the particles. The supplied solutions contain 10 mg/mL nanoplastics. The actual sizes of the 80 nm, 200 nm and 2.0 μm PS, PS-NH_2_ and PS-COOH beads were determined using SEM to be 81 ± 0.55 nm (PS), 85 ± 1.05 nm (PS-NH_2_) and 83 ± 0.62 nm (PS-COOH); 202 ± 0.89 nm (PS), 204 ± 2.05 nm (PS-NH_2_) and 207 ± 1.23 nm (PS-COOH); and 2.02 ± 0.29 μm (PS), 2.12 ± 0.23 μm (PS-NH_2_) and 2.08 ± 0.32 μm (PS-COOH), respectively (Fig. 1 and Additional file 1: Fig. S1).

DLS was used to quantify the hydrodynamic particle size and surface charge of nanoplastics using Zetasizer Nano ZS (Malvern)^19^. The 80 nm PS-COOH had a negative zeta potential (− 14.36 ± 0.55 mV), whereas 80 nm PS-NH_2_ beads had a positive zeta potential (8.71 ± 1.08 mV).

*E*. *coli* K12 and *B. subtilis* 168 were purchased from China General Microbiological Culture collection centre (CGMCC, China). *D*. *radiodurans* R1 was purchased from American Type Culture Collections (ATCC). All bacterial strains were grown under optimum growth conditions. *D*. *radiodurans* R1 strain was grown at 30 °C in TGY medium (0.5% tryptone, 0.1% glucose and 0.3% yeast extract) with aeration or on TGY plates supplemented with 1.5% Bacto-agar. *E*. *coli* K12 and *B. subtilis* were grown in Luria-Bertani (LB) broth (1.0% tryptone, 0.5% NaCl and 0.5% yeast extract) with aeration or on LB agar at 37 °C.

### Cell growth curve

All bacteria strains were cultivated to OD_600_ = 0.1. Then, 10 mL bacterial cultures were incubated with 20, 100 and 200 µg/mL PS-NH_2_ (80 nm) in a glass test tube (30 mL capacity) at 37 °C (*E*. *coli* and *B. subtilis*) or 30 °C (*D*. *radiodurans*) for 3 h. Bacterial cultures grown in the absence of PS-NH_2_ were used as the control. After the incubation, 100 µL cell cultures of different time points were spread on TGY or LB plate and incubated at 30 or 37 °C for 24 h. The colony forming units (CFUs) were counted. The growth curve was generated using the logarithm of CFU/mL versus the incubation time (h). The error bars represented the standard deviations from independent experiments performed in triplicate.

### Survival fractions of bacteria exposed to nanoplastics

Bacterial cells cultivated to OD_600_ = 1.0 were incubated with 100 µg/mL PS particles with different surface charges (neutral, positive and negative) and sizes (80 nm, 200 nm and 2 μm) for 3 h. For the survival of bacteria exposed to 80 nm PS-NH_2_ coated with lipids, the nanoparticles were first incubated with a lipid mixture containing phospholipids (3:1 of POPE:POPG) and fatty acids (4:1 of squalene acid:oleic acid) in the water phase. The bacterial cultures without PS particles were used as the control. After the exposure, 100 µL cell cultures were diluted with phosphate buffer (pH 7.0) in series by 10-fold and spread on TGY or LB plate for incubation at 30 or 37 °C before the counting of the colonies. The number of survival fractions was the ratio of the number of colonies from the treated cells to those from the control cells. The error bars represent the standard deviations obtained from independent experiments performed in triplicate.

### SEM and TEM

For SEM analysis, bacterial cells cultivated to OD_600_ = 1.0. The cells were collected by centrifugation at 2000 × g for 3 min and washed three times with deionized water. Then, 10 mL bacterial cells were incubated with 100 µg/mL nanoplastics (PS, PS-NH_2_ and PS-COOH with different particle sizes) for 3 or 12 h. The cells were collected by centrifugation at 2000 × g for 3 min and washed with deionized water thrice. Approximately 200 mg (wet weight) of bacteria cells were processed according to the method described previously [51]. The samples were fixed with 2.5% glutaraldehyde in phosphate buffer (pH 7.0) overnight. The dehydrated samples were coated with gold-palladium and observed using SEM (Hitachi, Japan).

For TEM analysis, the cell samples were fixed and dehydrated as described above, with the exception of the agar embedding step. Thin sections of the samples were stained with uranyl acetate for 15 min and observed via TEM (Hitachi, Japan). For the relative intracellular nanoplastics level assay, the nanoplastics in bacteria were calculated by counting the intracellular nanoplastics of five-independent cells in the TEM images, where the average numbers of nanoplastics in the cell cross section were used to represent the relative level of accumulated intracellular nanoplastics per cell. And the cells with destroyed cell membrane was not chosen for calculation.

For the observation of LCB by TEM, a drop of LCB solution was deposited onto a carbon-coated copper grid. The sample was then washed with ddH_2_O twice and dried completely in the air before observation.

### Confocal laser scanning microscopy

Confocal laser scanning microscopy was performed to determine the localisation and morphology of PS-NH_2_ in bacterial cells. The cell cultures were exposed to green fluorescently labelled PS-NH_2_ (80 nm) for 3 h, after which they were centrifuged and gently washed with phosphate buffer (pH 7.0). Bacterial cells in the absence of PS-NH_2_ were used as the control. Cell membrane stained with the Dil dye (red fluorescence) was detected at 549 nm excitation and 565 nm emission wavelengths, and the green fluorescently labelled PS-NH_2_ was detected at 505 nm excitation and 515 nm emission wavelengths under the confocal laser scanning microscope (Zeiss LSM510). Merged images indicated the overlap of the image of membrane lipid stained using the Dil and the image of PS-NH_2_.

### Coarse-grained models in computer simulation

For the coarse-grained nanoplastic Martini model, each polystyrene monomer was mapped onto four interaction beads: one corresponding to the position of the backbone atoms, and the other three representing the aromatic ring (Additional file 1: Fig. S7a). This mapping corresponded to the “A-mapping” scheme developed by Rossi et al.[52] that reproduced the best structural properties of polystyrene. The two outermost aromatic beads were designated as cross-linking particles. First, we constructed a pure polymer chain containing 100 styrene monomers (PS100). On the basis of PS100, we introduced the neutral, negative and positive groups to construct the three doped polymer chains, as shown in Additional file 1: Fig. S7b and c. The charged groups are evenly distributed on each polymer.

For the lipid bilayer models, two common phospholipids (POPE and POPG) in the cytoplasmic membrane of Gram-negative bacterium (*E. coli*) were used in our simulations with a molar ratio of 3:1 (POPE:POPG) [37]. We set up the cytoplasm membrane with surface dimensions of 40 nm × 40 and 80 nm × 80 nm (Additional file 1: Fig. S8); the membrane contained 4212 POPE and 1404 POPG (16,848 POPE and 5616 POPG, respectively). The membrane models were generated by using CHARMM-GUI (http://www.charmm-gui.org). Subsequently, the membrane was solvated and ionised with 0.15 M NaCl solution and then equilibrated for 1000 ns at 310 K and 1 atm. The final membrane structure was used for studying the interaction of the membrane with differentially charged nanoplastics.

### Molecular dynamics simulations

Molecular dynamics simulations on the coarse-grained models were performed in the NPT ensemble with periodic boundary conditions in all directions. The system temperature was maintained using the velocity-rescaled Berendsen thermostat [53]. The pressure was controlled using isotropic Parrinello-Rahman pressostat [54]. The force field parameters of nanoplastics were obtained from previous works [52, 55]. The interactions of the lipid membrane were described by the Martini force field [44, 56]. The long-range electrostatic interactions were treated with the particle mesh Ewald (PME) method [57]. The van der Waals (vdW) interactions were calculated with a cut off distance of 1.2 nm. All molecular dynamics simulations were conducted with the GROMACS package (version 5.1.4) [58]. Snapshots were rendered by the visual molecular dynamics (VMD) program [59].

### Interaction of PS-NH_2_ with membranes

Artificial lipid layer formation was performed using the previous method with some modifications [37]. The lipid layer was formed by pre-mixing phospholipids (POPE:POPG = 3:1) and fatty acids oil (squalene:oleic acid = 4:1) in the water phase. A drop of the lipid mixture, which was stained with Dil dye, was added into the PS-NH_2_ (2 μm) solution (20 µg/mL). The solution was vortexed and incubated at room temperature for 10 min. After the mixed solution was centrifuged and washed with deionised water thrice, the collected samples were analysed using the confocal laser scanning microscopy. Meanwhile, the samples that were not stained with Dil dye were analysed using SEM. For the interaction of PS-NH_2_ with a synthetic unilamellar liposome, the above-described lipid mixture in the water phase was ultrasonicated for 5 min to form liposome. The liposome solution was then mixed with PS-NH_2_ (2 μm; 20 µg/mL) and incubated at room temperature for 15 min. A macroscopic lipid layer (~ 5 mm) was formed by dropping the lipid and fatty acid oil mixture onto the surface of the water phase. Then, 1 µL nanoplastic solution (80 nm, 20 µg/mL) was gently dripped on the surface or near the edge of lipid membrane. The video and image were obtained by camera or confocal laser microscopy.

For the interactions between the lipid-coated bacteria and the PS-NH_2_, the bacteria cells coated with lipid layers were prepared according to the reported method with some modifications [50]. Bacteria (1.5 ml) sub-cultured for 3 h were collected, washed and resuspended in 1 ml of ice-cold phosphate solution containing 12.5 mM of CaCl_2_. DOPA (Avanti Polar Lipids, Alabaster, AL, USA) and cholesterol (Sangon, Shanghai, China) were dissolved in 5 ml of chloroform at a 4:1 molar ratio. Then, the mixed solution was dried by nitrogen, resulting in a lipid film. The obtained film was hydrated in 1 ml of bacterial solution and vortexed for 15 min to generate LCB. The LCB was treated with 100 µg/mL PS-NH_2_. Then, the morphological changes were observed by TEM. Intracellular ROS and survival fractions of the LCB were measured, respectively.

### Intracellular ROS detection

Intracellular ROS level was measured by using 2’,7’-dichlorofluorescein diacetate (DCFH-DA) as a molecular probe to detect ROS [60]. The DCFH-DA was hydrolysed into DCFH by intracellular esterase and then oxidised by intracellular ROS into DCF, which produced fluorescence. Cell cultures (OD_600_ = 1.0) were treated with 100 µg/mL PS nanoplastics for 30 min. Then, the cells were collected by centrifugation and washed with phosphate buffer (pH 7.5) thrice to remove extracellular probes. The images were observed using the confocal laser scanning microscope (Zeiss LSM510). The fluorescence was quantitated using a fluorescence spectrophotometer (SpectraMax M5) at an excitation wavelength of 485 nm and an emission wavelength of 525 nm.

### Statistical analysis

All experiments were performed with at least three independent replicates unless stated otherwise. Student’s *t* tests were used to assess the significance among results. *P* < 0.05 was considered significant.

## Supplementary Information

**Additional file 1: Fig. S1**. Characterization of differentially charged PS microplastics with the average sizes of 200 nm (a-c) and 2 μm (d-f). (a, d) Scanning electron microscopy (SEM) images of non-charged PS, positively charged PS (PS-NH_2_), and negatively charged PS (PS-COOH). Scale bars, 500 nm in Fig.S1a, and 5 μm in Fig.S1d. (b, e) Size distribution of the PS, PS-NH_2_, and PS-COOH as assessed using DLS in deionized water. (c,f) Stability of differentially charged PS microplastics in deionized water, TGY or LB medium. **Fig. S2.** Growth curves of *Bacillus subtilis* (a), *Escherichia coli* (b), and *Deinococcus radiodurans* (c) exposed to 80 nm PS and PS-COOH at 100 μg/mL for 24 h. Bacterial concentrations expressed as log10 (CFU/mL) of viable cells were measured at different growth times. **Fig. S3**. Growth curves of different bacteria under treatments of a mixture of the nanoplastics within cell debris collected from respective cell cultures exposed to 80 nm PS-NH_2_. (a) *B. subtilis*. (b) *E. coli*. (c) *D. radiodurans*. After incubating the bacterial cells with 100 μg/mL PS-NH_2_ (80 nm) for 24 h, the nanoplastics within cell debris were collected from the cell lysate by centrifuge (10000 g). Then, the mixture was added to the fresh culture of each bacterium to monitor cell growth. Bacteria concentrations expressed as log10 (CFU/mL) of viable cells were measured at different growth times. **Fig. S4**. PS-NH_2_ induced influx of SYTOX green into *B. subtilis* cells. The SYTOX green uptake assay for measuring membrane permeability was following the methods as described previously (T. Vineeth Kumar, et al., Animal biotechnology, 2021,32(2):137–146; Evelien Gerits, et al., Clinical and experimental dental research, 2017, 3(2): 69–76). The SYTOX green is impermeable to cells with intact inner membrane and enters the cells only in case of membrane damage. Bacterial cell samples were prepared as per the above experiments. The SYTOX green (2 μM) and DAPI (50 ng/ml) was added and incubated. Cell stained with the SYTOX was detected at 502 nm excitation and 525 nm emission wavelengths, and the DAPI was detected at 405 nm excitation and 488 nm emission wavelengths under the confocal laser scanning microscope (Zeiss LSM510). The first vertical panel shows the blue fluorescence of DAPI, which is used to visualize all the bacterial cells. The second vertical panel represents SYTOX signal, which is used to indicate the uptake of SYTOX green in the envelope-damaged cells induced by 80 nm PS-NH_2_ (100 μg/mL). Merged images indicate the combined signals of DAPI and SYTOX. Control, images of *B. subtilis* cells without the treatment of PS-NH_2_. Scale bars correspond to 1 μm. **Fig. S5**. Amplified TEM images of the cell morphology exposed to PS-NH_2_ (80 nm). *E. coli*, *D. radiodurans*, and *B. subtilis* cells at OD_600_ of 1.0 were incubated with 100 μg/mL PS-NH_2_ (80 nm) for 3 h at 37 °C or 30 °C, respectively. Scale bars, 500 nm. **Fig. S6.** SEM and TEM images of cell morphology of *E. coli*, *B. subtilis* and *D. radiodurans* exposed to 80 nm PS or PS-COOH for 12 h. Bacterial cells at OD_600_ of 1.0 were incubated with 100 μg/mL 80 nm PS and PS-COOH for 12 h at 37 °C or 30 °C, respectively. Scale bars in SEM images, 1 μm; Scale bars in TEM images, 500 nm. **Fig S7**. The coarse-grained representation of the PS nanoplastics. (a) The structural formula (left) and carse-grained model (right) of polystyrene (trimer). (b) Neutral, negatively charged, and positively charged doped polymer chain structure, m=84, n=16. (c) Surface charge distribution of three types of nanoplastics (16 nm). Red spot, negative charge; Blue spot, positive charge. **Fig. S8**. The coarse-grained representation of *E. coli* cytoplasm membrane. (a) Coarse-grained models of POPE and POPG: colors indicate the PE headgroups (blue), the PG headgroups (pink), the glycerol ester moieties (purple), the tail groups (green). The snapshots of the membrane lipid bilayer (POPE: POPG=3:1) for 40nm × 40 nm area (b) and 80nm × 80nm area (c) are shown. **Fig. S9**. The definition of insertion depth d_z_ (a) and lipid tail tilt angle (b), the planar bilayer normal is in the z direction. The insertion depth d_z_ is defined as the distance between the highest atom in the upper membrane and the lowest atom in the lower membrane along with the z direction. **Fig S10**. Representative snapshots of the binding of nanoplastics to the mimic cytoplasm membrane and time-dependent insertion depth of the negatively charged, neutral, and positively charged PS nanoplastics (16 nm). (a) Simulation 2. (b) Simulation 3. **Fig. S11**. Temperature effects on the translocation capacity and cytotoxicity of positively charged nanoplastics on bacterial cells. (a) Representative snapshots of the binding of positively charged PS-NH_2_ (16 nm) to the mimic cytoplasm membrane at different temperatures. (b) Time-dependent insertion depth of PS-NH_2_ at different temperatures. (c) Survive fractions of different bacteria exposed to PS-NH_2_ (80nm) for 3 h at different temperatures. BS, *B. subtilis*; EC, *E. coli*; DR, *D. radiodurans*. **Fig. S12**. Experimental diagram of the effect of nanoplastics on the artificial lipid membrane.A macro artificial lipid membrane (radius ≈ 0.5 cm) was constructed by dripping a drop of the mixture of phospholipids and fatty acids on the surface of deionized water in a plate. And a drop of 80 nm PS with different charges was dripped from the top (1) or the side (2) of the artificial lipid membrane. The video and images were obtained by a camera or confocal laser scanning microscopy.

**Additional file 2: Movie 1**. Front view of the coarse-grained molecular simulations of the PS-NH_2_ uptake process by the membrane bilayers. The penetration of PS-NH_2_ led to gradual membrane invagination, the final internalisation of the lipid-coated nanoplastics around 1000 ns, and a finally closed transient pore.

**Additional file 3: Movie 2**. Top view of the coarse-grained molecular simulations of the PS-NH_2_ uptake process by the membrane bilayers. The penetration of PS-NH_2_ led to gradual membrane invagination, the final internalisation of the lipid-coated nanoplastics around 1000 ns, and a finally closed transient pore.

**Additional file 4: Movie 3**. PS-NH_2_ imposes surface tension leading to morphology changes of the macro artificial lipid membrane. 1 μL PS (80 nm) with different charges was dripped from the top of the artificial lipid membrane. +, PS-NH_2_; -, PS-COOH; 0, neutral PS.

## Data Availability

The datasets generated during and/or analyzed during the current study are available from the corresponding author on reasonable request.
