# Supplementary material for: Distinct lipid membrane interaction and uptake of differentially charged nanoplastics in bacteria
Source: J Nanobiotechnology. 2022 Apr 15;20:191. doi: 10.1186/s12951-022-01321-z (PMC9011954; doi:10.1186/s12951-022-01321-z)

**Distinct lipid membrane interaction and uptake of differentially charged nanoplastics in bacteria**

Shang Dai^a, 1^, Rui Ye^a, 1^, Jianxiang Huang^a^, Binqiang Wang^a^, Zhenming Xie^a^, Xinwen Ou^a^, Ning Yu^a^, Cheng Huang^a^, Yuejin Hua^*a^, Ruhong Zhou^* a, b^, Bing Tian^*a^

^a^ College of Life Sciences, Department of Physics, and Institute of Quantitative Biology, Zhejiang University, Hangzhou, China;

^b^ Department of Chemistry, Columbia University, New York, NY 10027, United States

^*^Corresponding author: Bing Tian, Ruhong Zhou and Yuejin Hua

Email: tianbing@zju.edu.cn; [rhzhou@zju.edu.cn](mailto:rhzhou@zju.edu.cn); [yjhua@zju.edu.cn](mailto:yjhua@zju.edu.cn)

**Fig. S1 Characterization of differentially charged PS microplastics with the average sizes of 200 nm (a-c) and 2 μm (d-f)**. (a, d) Scanning electron microscopy (SEM) images of non-charged PS, positively charged PS (PS-NH_2_), and negatively charged PS (PS-COOH). Scale bars, 500 nm in Fig.S1a, and 5 μm in Fig.S1d. (b, e) Size distribution of the PS, PS-NH_2,_ and PS-COOH as assessed using DLS in deionized water. (c,f) Stability of differentially charged PS microplastics in deionized water, TGY or LB medium.

**
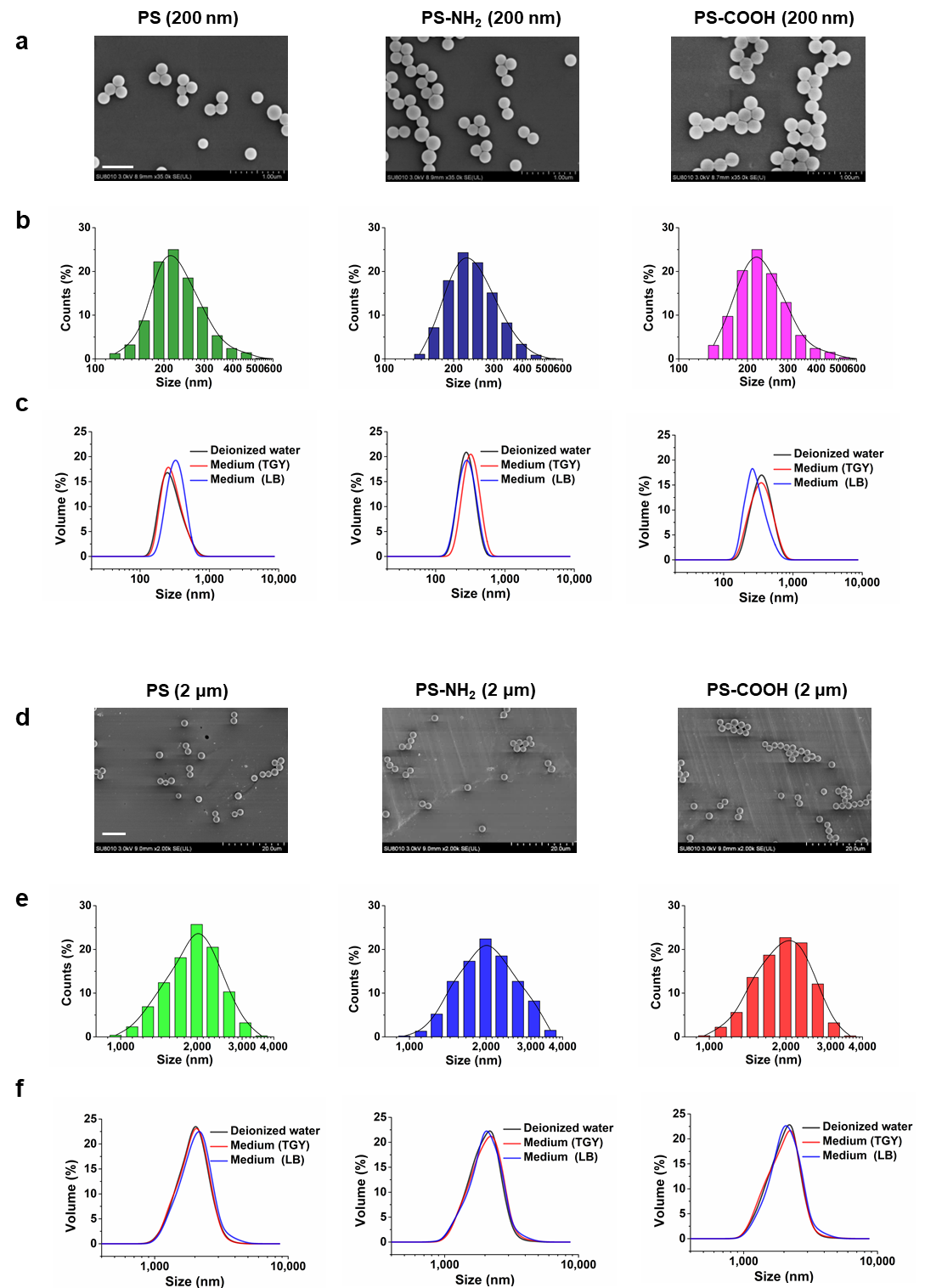
**

**Fig. S2. Growth curves of *Bacillus subtilis* (a)*, Escherichia coli* (b), and *Deinococcus radiodurans* (c) exposed to 80 nm PS and PS-COOH at 100 μg/mL for 24 h**. Bacterial concentrations expressed as log_10_ (CFU/mL) of viable cells were measured at different growth times.


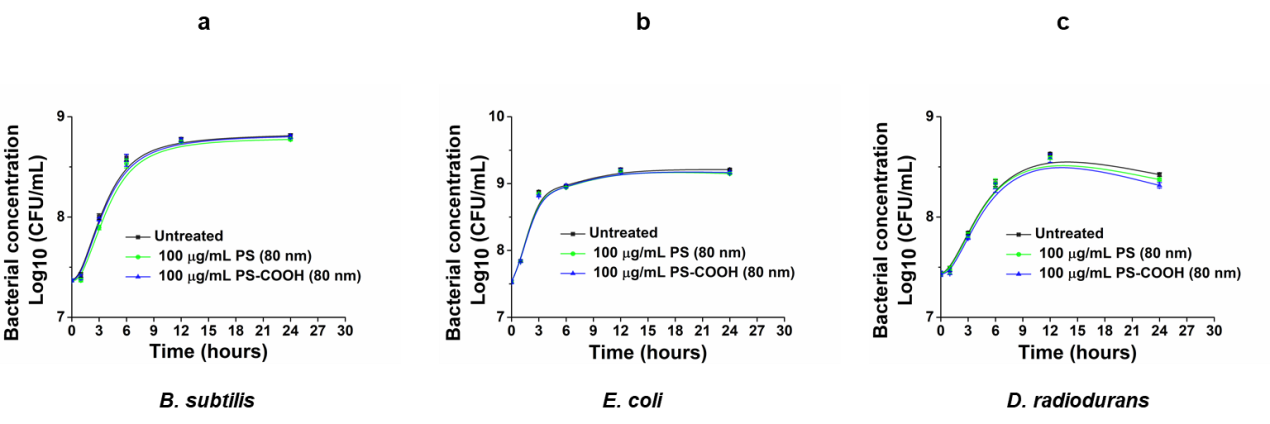


**Fig. S3 Growth curves of different bacteria under treatments of a mixture of the nanoplastics within cell debris collected from respective cell cultures exposed to 80 nm PS-NH_2_.** (a) *B*. *subtilis*. (b) *E*.*coli*. (c) *D*. *radiodurans*. After incubating the bacterial cells with 100 μg/mL PS-NH_2_ (80 nm) for 24 h, the nanoplastics within cell debris were collected from the cell lysate by centrifuge (10000 *g*). Then, the mixture was added to the fresh culture of each bacterium to monitor cell growth. Bacteria concentrations expressed as log_10_ (CFU/mL) of viable cells were measured at different growth times.


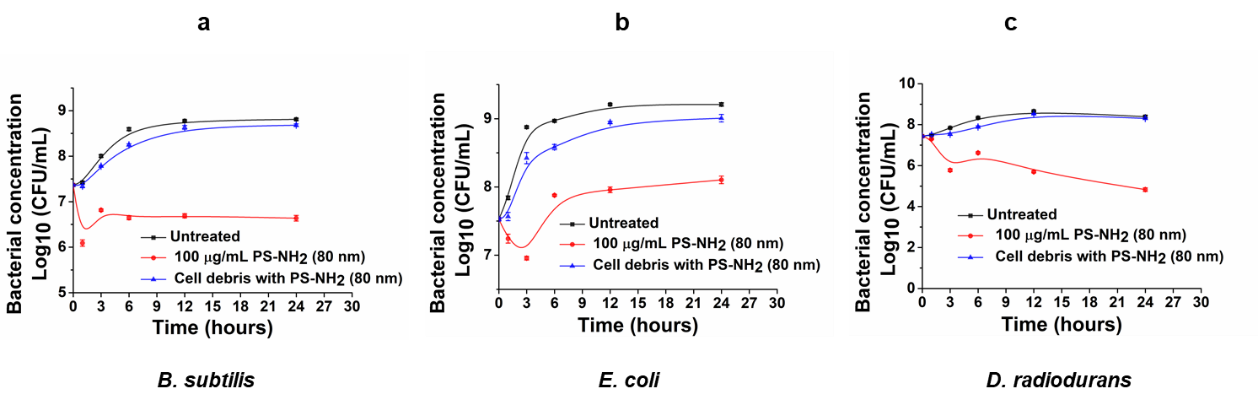


**Fig. S4 PS-NH_2_ induced influx of SYTOX green into *B. subtilis* cells.** The SYTOX green uptake assay for measuring membrane permeability was following the methods as described previously (T. Vineeth Kumar, *et al*., *Animal biotechnology*, 2021,*32*(2):137–146; Evelien Gerits, *et al*., *Clinical and experimental dental research*, 2017, *3*(2): 69–76). The SYTOX green is impermeable to cells with intact inner membrane and enters the cells only in case of membrane damage. Bacterial cell samples were prepared as per the above experiments. The SYTOX green (2 μM) and DAPI (50 ng/ml) was added and incubated. Cell stained with the SYTOX was detected at 502 nm excitation and 525 nm emission wavelengths, and the DAPI was detected at 405 nm excitation and 488 nm emission wavelengths under the confocal laser scanning microscope (Zeiss LSM510). The first vertical panel shows the blue fluorescence of DAPI, which is used to visualize all the bacterial cells. The second vertical panel represents SYTOX signal, which is used to indicate the uptake of SYTOX green in the envelope-damaged cells induced by 80 nm PS-NH_2_ (100 μg/mL). Merged images indicate the combined signals of DAPI and SYTOX. Control, images of *B. subtilis* cells without the treatment of PS-NH_2_. Scale bars correspond to 1 μm.


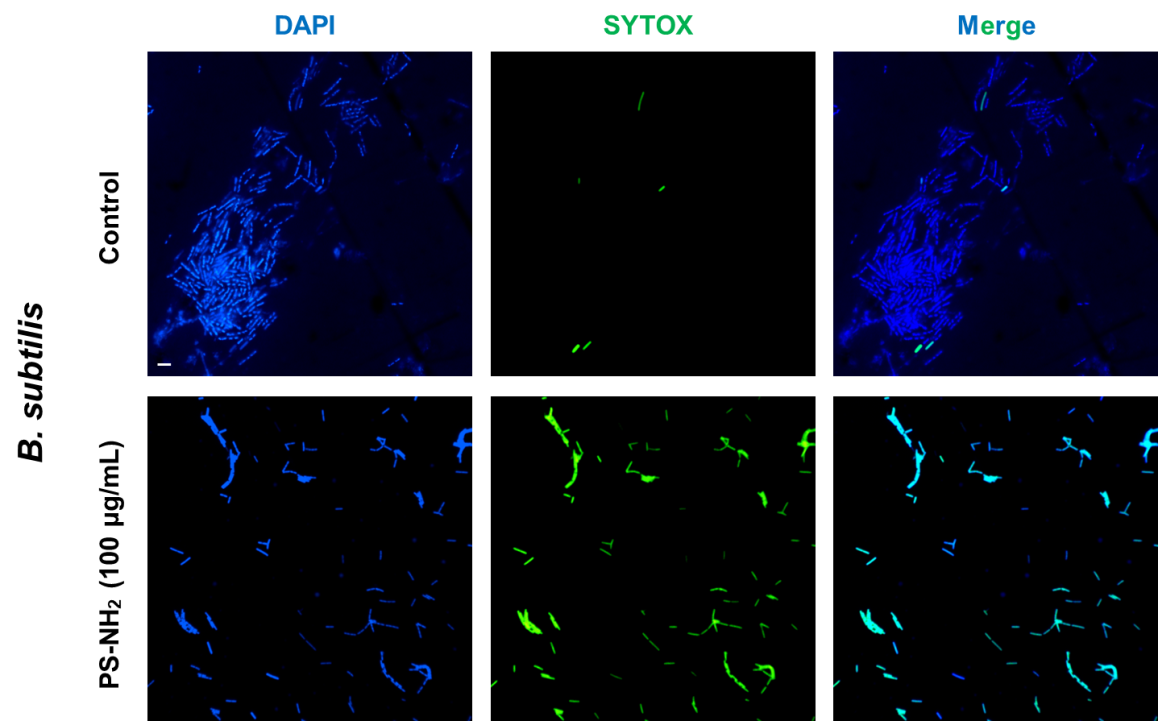


**Fig. S5. Amplified TEM images of the cell morphology exposed to PS-NH_2_ (80 nm).** *E*. *coli*, *D*. *radiodurans,* and *B*. *subtilis* cells at OD_600_ of 1.0 were incubated with 100 μg/mL PS-NH_2_ (80 nm) for 3 h at 37 ^o^C or 30 ^o^C, respectively. Scale bars, 500 nm.


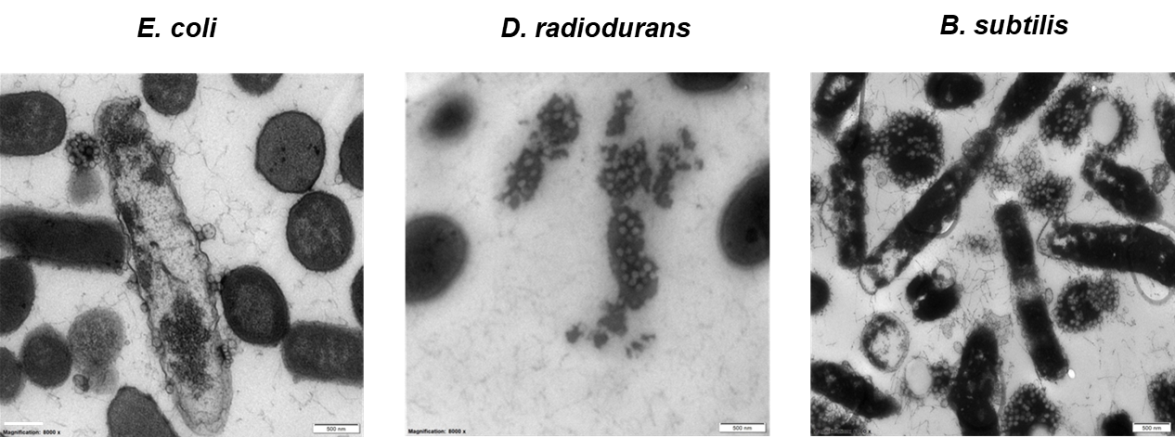


**Fig. S6 SEM and TEM images of** **cell morphology of *E. coli, B. subtilis and D. radiodurans* exposed to 80 nm PS or PS-COOH for 12 h.** Bacterial cells at OD_600_ of 1.0 were incubated with 100 μg/mL 80 nm PS and PS-COOH for 12 h at 37 ^o^C or 30 ^o^C, respectively. Scale bars in SEM images, 1 μm; Scale bars in TEM images, 500 nm.

**
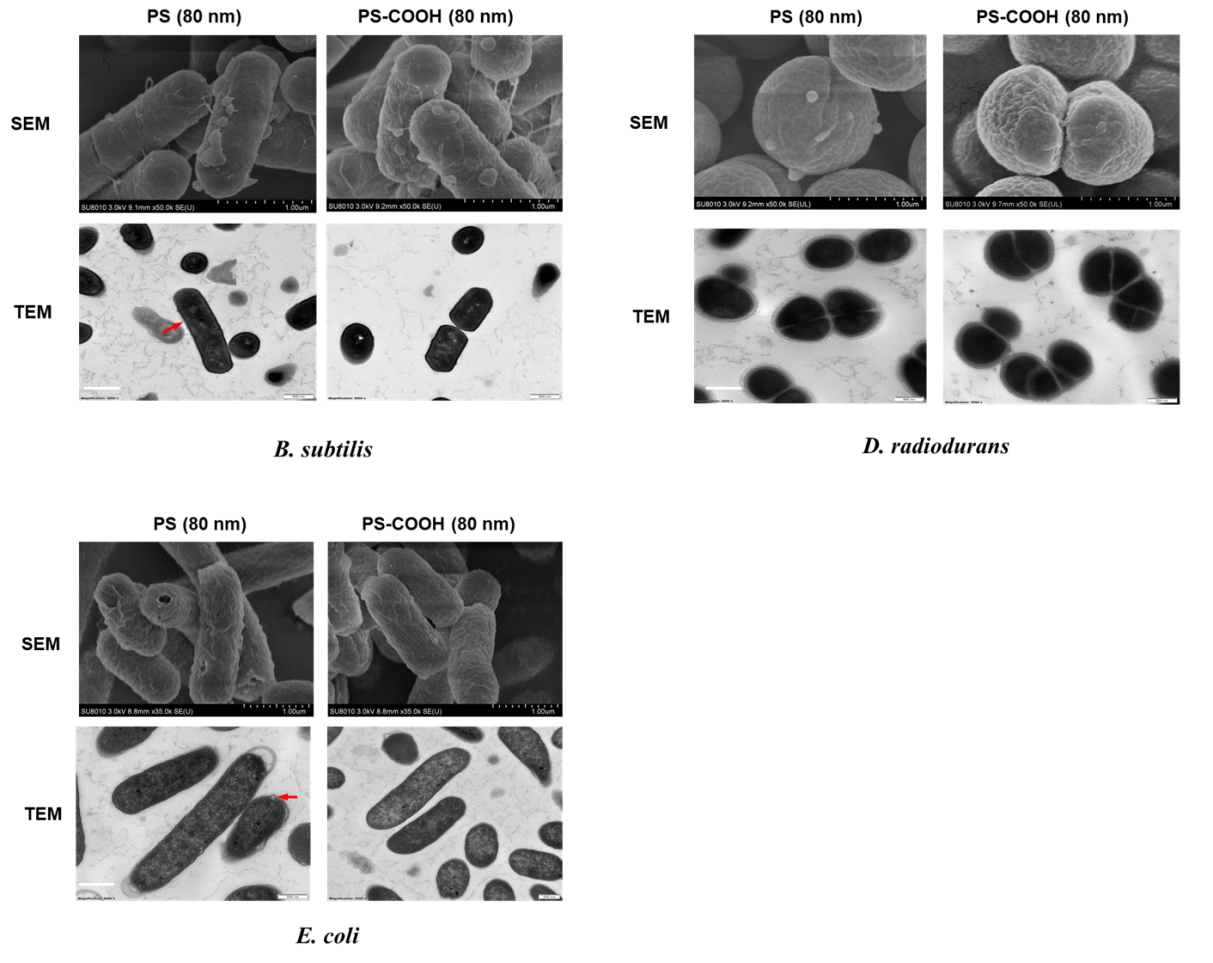
**

**Fig S7.** The coarse-grained representation of the PS nanoplastics. (a) The structural formula (left) and carse-grained model (right) of polystyrene (trimer). (b) Neutral, negatively charged, and positively charged doped polymer chain structure, m=84, n=16. (c) Surface charge distribution of three types of nanoplastics (16 nm). Red spot, negative charge; Blue spot, positive charge.


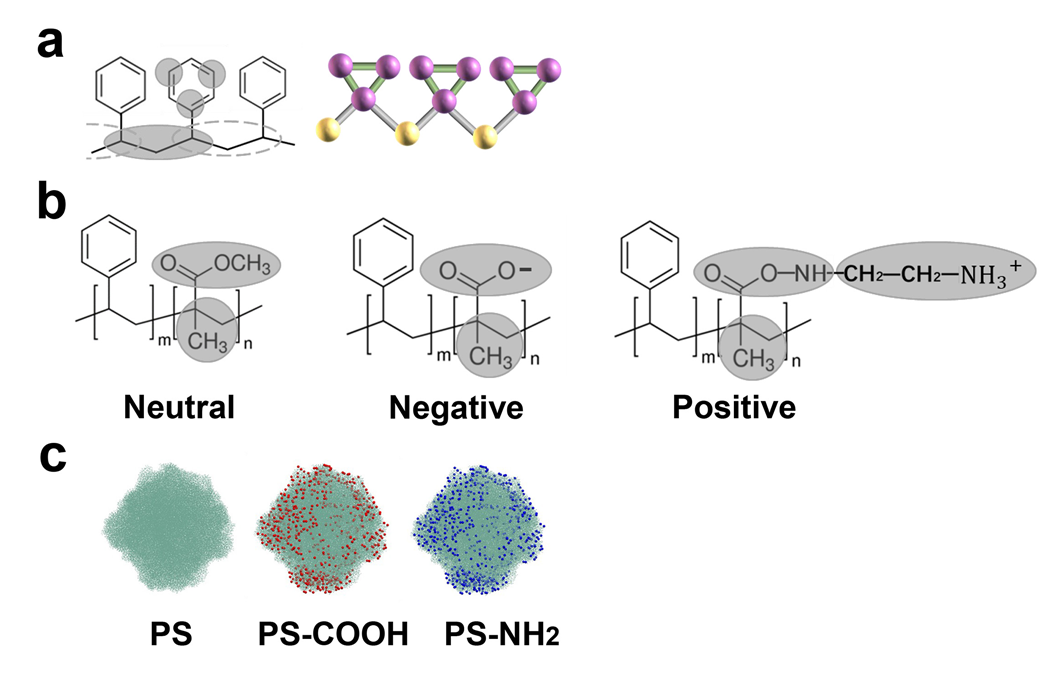


**Fig. S8.** The coarse-grained representation of *E. coli* cytoplasm membrane. (a) Coarse-grained models of POPE and POPG: colors indicate the PE headgroups (blue), the PG headgroups (pink), the glycerol ester moieties (purple), the tail groups (green). The snapshots of the membrane lipid bilayer (POPE: POPG=3:1) for 40nm × 40 nm area (b) and 80nm × 80nm area (c) are shown.


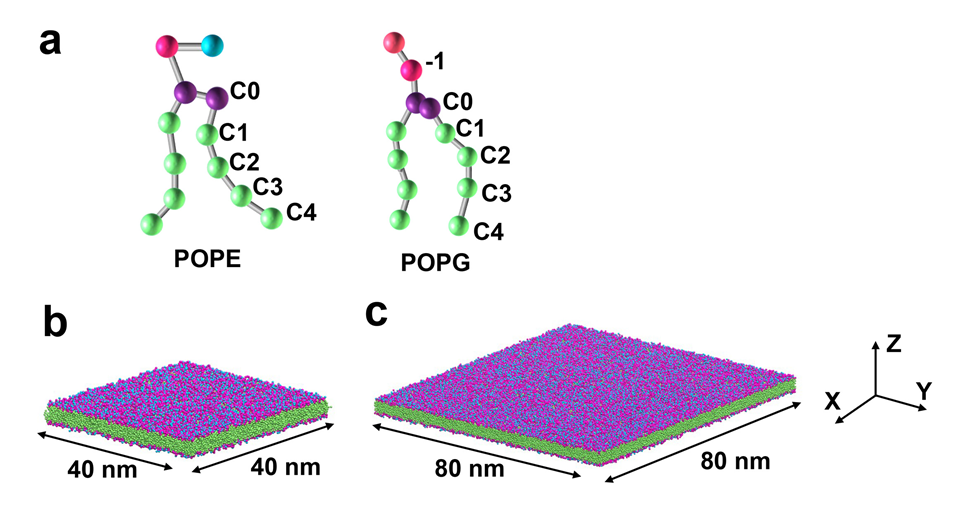


**Fig. S9.** The definition of insertion depth d_Z_ (a) and lipid tail tilt angle (b), the planar bilayer normal is in the z direction. The insertion depth d_z_ is defined as the distance between the highest atom in the upper membrane and the lowest atom in the lower membrane along with the z direction.


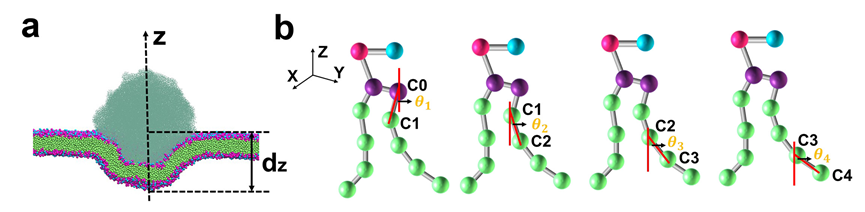


**Fig S10.** Representative snapshots of the binding of nanoplastics to the mimic cytoplasm membrane and time-dependent insertion depth of the negatively charged, neutral, and positively charged PS nanoplastics (16 nm). (a) Simulation 2. (b) Simulation 3.


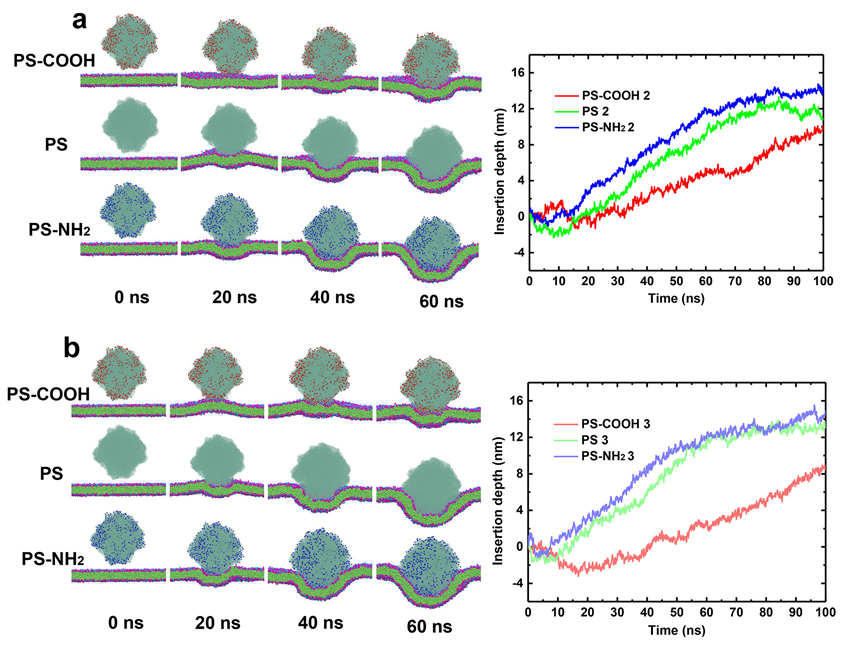


**Fig. S11 Temperature effects on the translocation capacity and cytotoxicity of positively charged nanoplastics on bacterial cells.** (a) Representative snapshots of the binding of positively charged PS-NH_2_ (16 nm) to the mimic cytoplasm membrane at different temperatures. (b) Time-dependent insertion depth of PS-NH_2_ at different temperatures. (c) Survive fractions of different bacteria exposed to PS-NH_2_ (80nm) for 3 h at different temperatures. BS, *B*. *subtilis*; EC,  *E*. *coli*; DR, *D*. *radiodurans*.

**
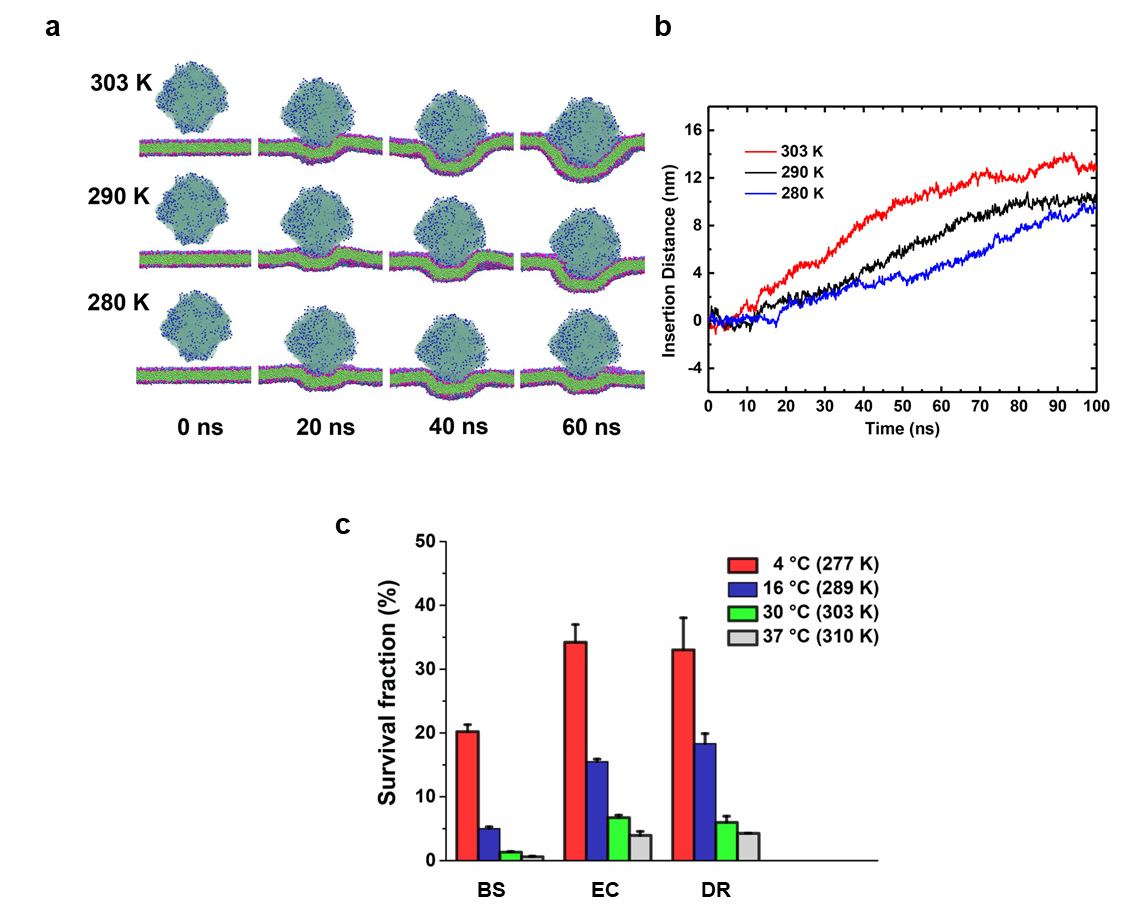
**

**Fig. S12 Experimental diagram of the effect of nanoplastics on the artificial lipid membrane.**

A macro artificial lipid membrane (radius ≈ 0.5 cm) was constructed by dripping a drop of the mixture of phospholipids and fatty acids on the surface of deionized water in a plate. And a drop of 80 nm PS with different charges was dripped from the top (1) or the side (2) of the artificial lipid membrane. The video and images were obtained by a camera or confocal laser scanning microscopy.


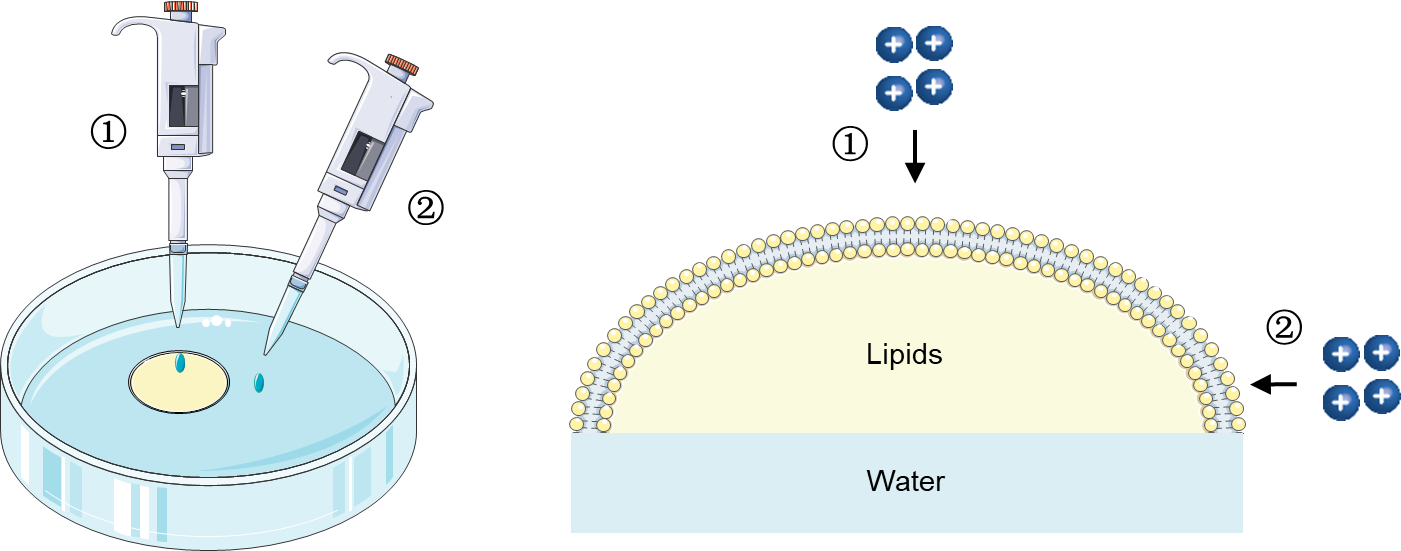

Supplement: Supplementary file 1 — Additional file 1: Fig. S1. Characterization of differentially charged PS microplastics with the average sizes of 200 nm (a-c) and 2 μm (d-f). (a, d) Scanning electron microscopy (SEM) images of non-charged PS, positively charged PS (PS-NH2), and negatively charged PS (PS-COOH). Scale bars, 500 nm in Fig.S1a, and 5 μm in Fig.S1d. (b, e) Size distribution of the PS, PS-NH2, and PS-COOH as assessed using DLS in deionized water. (c,f) Stability of differentially charged PS microplastics in deionized water, TGY or LB medium. Fig. S2. Growth curves of Bacillus subtilis (a), Escherichia coli (b), and Deinococcus radiodurans (c) exposed to 80 nm PS and PS-COOH at 100 μg/mL for 24 h. Bacterial concentrations expressed as log10 (CFU/mL) of viable cells were measured at different growth times. Fig. S3. Growth curves of different bacteria under treatments of a mixture of the nanoplastics within cell debris collected from respective cell cultures exposed to 80 nm PS-NH2. (a) B. subtilis. (b) E. coli. (c) D. radiodurans. After incubating the bacterial cells with 100 μg/mL PS-NH2 (80 nm) for 24 h, the nanoplastics within cell debris were collected from the cell lysate by centrifuge (10000 g). Then, the mixture was added to the fresh culture of each bacterium to monitor cell growth. Bacteria concentrations expressed as log10 (CFU/mL) of viable cells were measured at different growth times. Fig. S4. PS-NH2 induced influx of SYTOX green into B. subtilis cells. The SYTOX green uptake assay for measuring membrane permeability was following the methods as described previously (T. Vineeth Kumar, et al., Animal biotechnology, 2021,32(2):137–146; Evelien Gerits, et al., Clinical and experimental dental research, 2017, 3(2): 69–76). The SYTOX green is impermeable to cells with intact inner membrane and enters the cells only in case of membrane damage. Bacterial cell samples were prepared as per the above experiments. The SYTOX green (2 μM) and DAPI (50 ng/ml) was added and incubated. Cell [file 12951_2022_1321_MOESM1_ESM.docx]
